# Epithelial-mesenchymal transition: a hallmark in pancreatic cancer stem cell migration, metastasis formation, and drug resistance

**DOI:** 10.20517/2394-4722.2020.55

**Published:** 2020-09-27

**Authors:** Ahmad R. Safa

**Affiliations:** Department of Pharmacology and Toxicology, Indiana University School of Medicine, Indianapolis, IN, 46202, USA.

**Keywords:** Pancreatic cancer, cancer stem cells, epithelial-mesenchymal transition, metastasis, drug resistance

## Abstract

Metastasis, tumor progression, and chemoresistance are the major causes of death in patients with pancreatic ductal adenocarcinoma (PDAC). Tumor dissemination is associated with the activation of an epithelial-to-mesenchymal transition (EMT) process, a program by which epithelial cells lose their cell polarity and cell-to-cell adhesion, and acquire migratory and invasive abilities to become mesenchymal stem cells (MSC). These MSCs are multipotent stromal cells capable of differentiating into various cell types and trigger the phenotypic transition from an epithelial to a mesenchymal state. Therefore, EMT promotes migration and survival during cancer metastasis and confers stemness features to particular subsets of cells. Furthermore, a major problem limiting our ability to treat PDAC is the existence of rare populations of pancreatic cancer stem cells (PCSCs) or cancer-initiating cells in pancreatic tumors. PCSCs may represent sub-populations of tumor cells resistant to therapy which are most crucial for driving invasive tumor growth. These cells are capable of regenerating the cellular heterogeneity associated with the primary tumor when xenografted into mice. Therefore, the presence of PCSCs has prognostic relevance and influences the therapeutic response of tumors. PCSCs express markers of cancer stem cells (CSCs) including CD24, CD133, CD44, and epithelial specific antigen as well as the drug transporter ABCG2 grow as spheroids in a defined growth medium. A major difficulty in studying tumor cell dissemination and metastasis has been the identification of markers that distinguish metastatic cancer cells from cells that are normally circulating in the bloodstream or at sites where these cells metastasize. Evidence highlights a linkage between CSC and EMT. In this review, The current understanding of the PCSCs, signaling pathways regulating these cells, PDAC heterogeneity, EMT mechanism, and links between EMT and metastasis in PCSCs are summarised. This information may provide potential therapeutic strategies to prevent EMT and trigger CSC growth inhibition and cell death.

## INTRODUCTION

Pancreatic ductal adenocarcinoma (PDAC) is the fourth leading cause of cancer-related death in the world with a low probability of early diagnosis^[1]^. KRAS, CDKN2A, TP53, and SMAD4 are frequently mutated genes that define the genetic landscape of PDAC. PDAC is one of the most lethal cancers due to its high metastatic potential and delayed detection^[2,3]^. The median survival time following diagnosis remains at less than 6 months, with an overall survival rate of less than 4%^[1–3]^. Gemcitabine (GEM) treatment has only increased the median survival of PDAC patients from 3–4 months to 6–7 months^[4–6]^. Recent evidence shows that using FOLFIRINOX (leucovorin, fluorouracil, irinotecan, oxaliplatin) in PDAC patients was more effective than GEM as it demonstrated longer survival in pancreatic cancer patients (11.1 months *vs.* 6.8 months)^[6,7]^. Resistance to apoptosis is a common feature of PDAC and a major reason why this devastating disease is resistant to various treatment strategies including GEM and FOLFIRINOX^[8]^. Another major problem limiting our ability to treat PDAC is the existence of rare populations of pancreatic cancer stem cells (PCSCs) or cancer-initiating cells in pancreatic tumors; PCSCs may represent sub-populations of tumor cells resistant to therapy which are most crucial for driving invasive tumor growth^[9–13]^. The PCSCs express a wide array of markers such as CD44, CD24, epithelial specific antigen (ESA), CD133, c-mesenchymal to epithelial transition (c-MET), CXCR4, PD2/Paf1, and ALDH1^[14–17]^. These cells are capable of regenerating the cellular heterogeneity associated with the primary tumor when xenografted into mice^[13–17]^. Therefore, the presence of PCSCs has prognostic relevance and influences the therapeutic response of tumors.

Metastasis is the major cause of high PDAC mortality. As Mu *et al.*^[15]^ recently described, tumor progression is driven by the cross-interaction between tumor cells, primarily cancer stem cells (CSCs) (or cancer-initiating cells) and surrounding stromal cells as well as distant organs, in which tumor-derived extracellular vesicles (TEX) play a major and important role. Mu *et al.*^[15]^ report that the PCSC markers Tspan8, alpha6beta4, CD44v6, CXCR4, LRP5/6, LRG5, claudin7, EpCAM, and CD133, participate in a metastatic cascade at various steps, often via PDAC CSC-TEX.

In this review, the models of CSCs in PDAC and their cell-intrinsic and - extrinsic regulatory pathways are described. Insights into the heterogeneity of cell sub-populations of PDAC, plasticity, cancer stemness, and the involvement of epithelial-mesenchymal transition (EMT), which participates in metastasis are highlighted. These properties may account for the unsuccessful clinical trials that test therapeutics designed to directly target CSCs.

## PCSCS

CSCs from epithelial tissues were first identified in breast cancer in 2003 by Al-Hajj *et al.*^[18]^, who found that a distinct sub-population of cancer cells expressing CD44+CD24−/low ESA+ develop into tumors in immunodeficient mice. In PDAC, the presence of CSCs was reported in 2007 by Shah *et al.*^[19]^ who demonstrated that CD44+CD24+ESA+ cells exhibit high tumorigenic potential.

**Figure 1.**
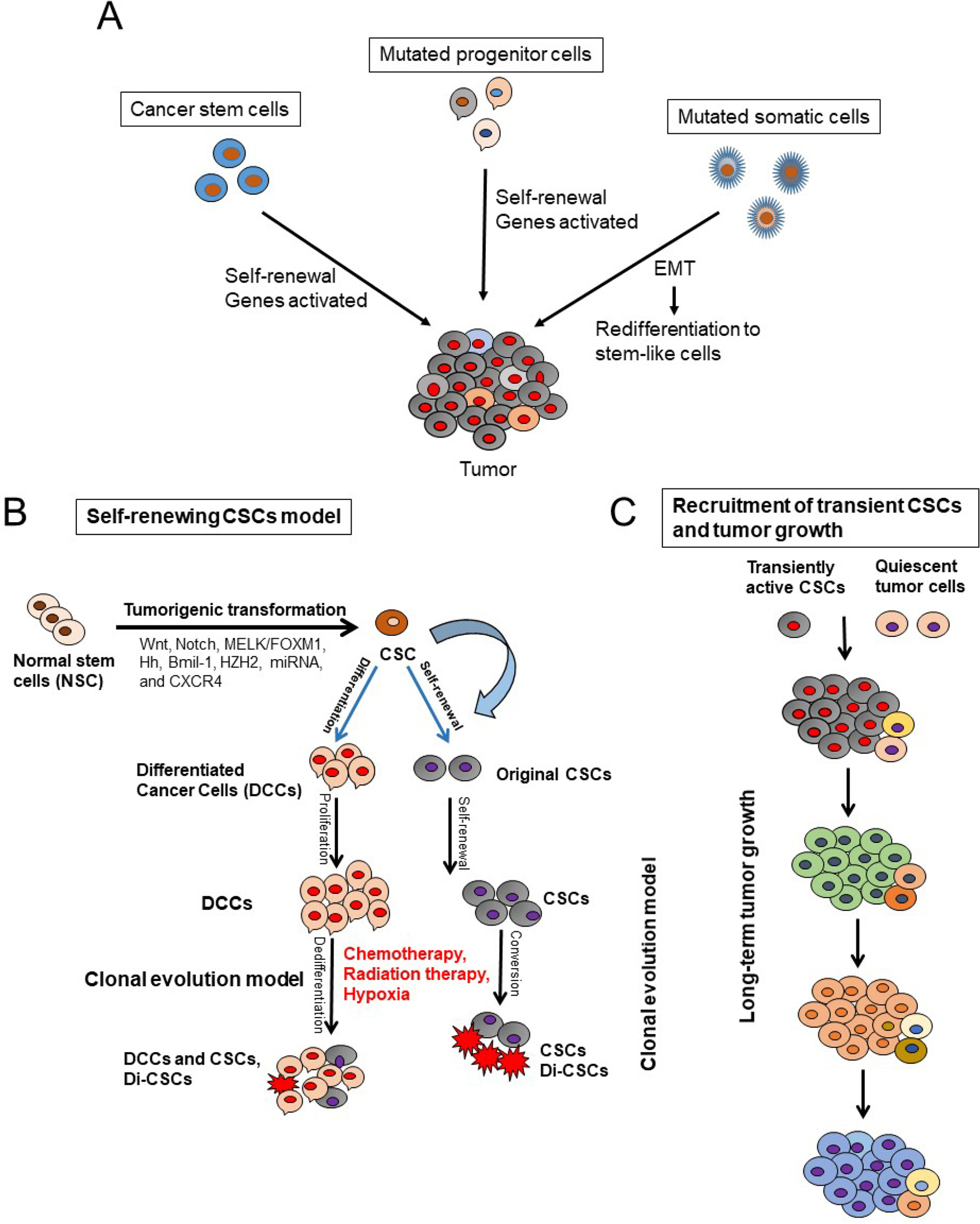
Models that explain pancreatic cancer tumor development and roles of cancer stem cells (CSCs) in tumor initiation and progression. A: stochastic model shows that every cell has the potential to be the tumor initiator; B: CSC model shows that CSCs originate from normal stem cells (NSCs) through mutations and tumorigenic transformation of several potential pathways including Hh: hedgehog, epithelial-to-mesenchymal transition (EMT), and the reverse process mesenchymal-to-epithelial transition (MET). CSCs are also generated by de-differentiation of differentiated malignant cells by chemotherapeutic agents, ionizing radiation, and hypoxia. CSCs and drug-induced CSCs (Di-CSCs) are enriched following conventional chemotherapy treatment; C: the model of long-term tumor growth in PDAC states that a succession of CSC or tumor initiating cells (TIC) clones and drives tumor progression in serial xenotransplantation. Individual TICs contribute to tumor formation transiently and generate mainly non-tumorigenic progeny expressing little or no self-renewal

Two models are proposed to describe the origin of CSCs [Figure 1] and their role in tumorigenesis^[20–22]^. The first one is the hierarchical model in which the CSCs represent a small distinct sub-population within the tumor with capacity for self-renewals and also the ability to differentiate into progeny cells; the progeny cells then proliferate to initiate and sustain tumor development and progression. Therefore, this model indicates that in the clinical setting, eradication of CSCs may prevent tumor recurrence.

The second model is stochastic model in which every cell within a tumor is capable of promoting tumor initiation and progression^[21]^. Furthermore, the heterogeneity within the tumor cell population is due to accumulated genetic mutations^[21]^. These CSCs models may express plasticity in the presence of tumor microenvironmental stimuli including oxidative and nutritional stress, low oxygen tension, and cytotoxic drugs to which the tumor can be subjected to^[16,21–25]^. These factors influence the inter-conversion of a non-CSC population to a CSCs^[21,22]^ [Figure 1]. Previous studies have demonstrated that extracellular matrix components like collagen can influence clonogenic tumor growth, tumor initiation, and invasion of PDAC due to activation of the FAK signaling pathway^[26,27]^. Additionally, a hypoxic tumor microenvironment is known to promote the conversion of non-CSC to CSC populations in PDAC^[8]^. These data corroborate with observations that the CSCs within the tumor are the cells’ survival advantage, capable of living under stress conditions and to express resistance to cancer therapies.

Accumulated evidence obtained through large-scale genomic studies and single cell RNA sequencing analysis has indicated the existence of CSCs in hematological malignancies and solid tumors including PDAC, breast cancer, malignant glioma, prostate cancer, non-small cell lung cancer, colorectal cancer, and hepatocellular carcinoma^[28–37]^. Substantial *in vitro* and *in vivo* studies have provided convincing evidence supporting CSCs as the critical cause of cancer initiation, growth, metastasis, and treatment resistance in cancers^[20–24]^. CSCs were also found to exhibit characteristics of an EMT, a process known to enable cancer cell dissemination and metastasis to other organs^[35–41]^. Additionally, significant and convincing evidence shows that inducing EMT in cancer cells confers cancer stem cell-like characteristics, which promote their metastatic and tumor-initiating abilities^[37,38]^. While PCSCs self-renewal is essential in the progression, migration, and metastasis of PDAC, clonal evolution and plasticity of PCSCs within PDAC tumors are not well understood^[38–46]^. In this regard, Ball *et al.*^[47]^ showed that long-term progression of PDAC in serial xenotransplantation happens by a succession of transiently active PCSCs producing tumor cells in a temporally restricted manner with little overlap between subsequent xenograft generations. Therefore, the clonal PCSC activity in PDAC differs from the continuous activity of limited numbers of self-renewing PCSCs with a defined and rigid cellular hierarchy. Indeed, PCSCs heterogeneity and plasticity make therapeutic targeting of PCSCs very difficult and challenging^[24]^. Furthermore, several investigations have suggested that CSCs exist in dynamic equilibrium with more differentiated cancer cells via bi-directional regeneration or interconversion of differentiated cancer cells to CSCs, caused by various factors^[23,24,48,49,50]^.

**Figure 2.**
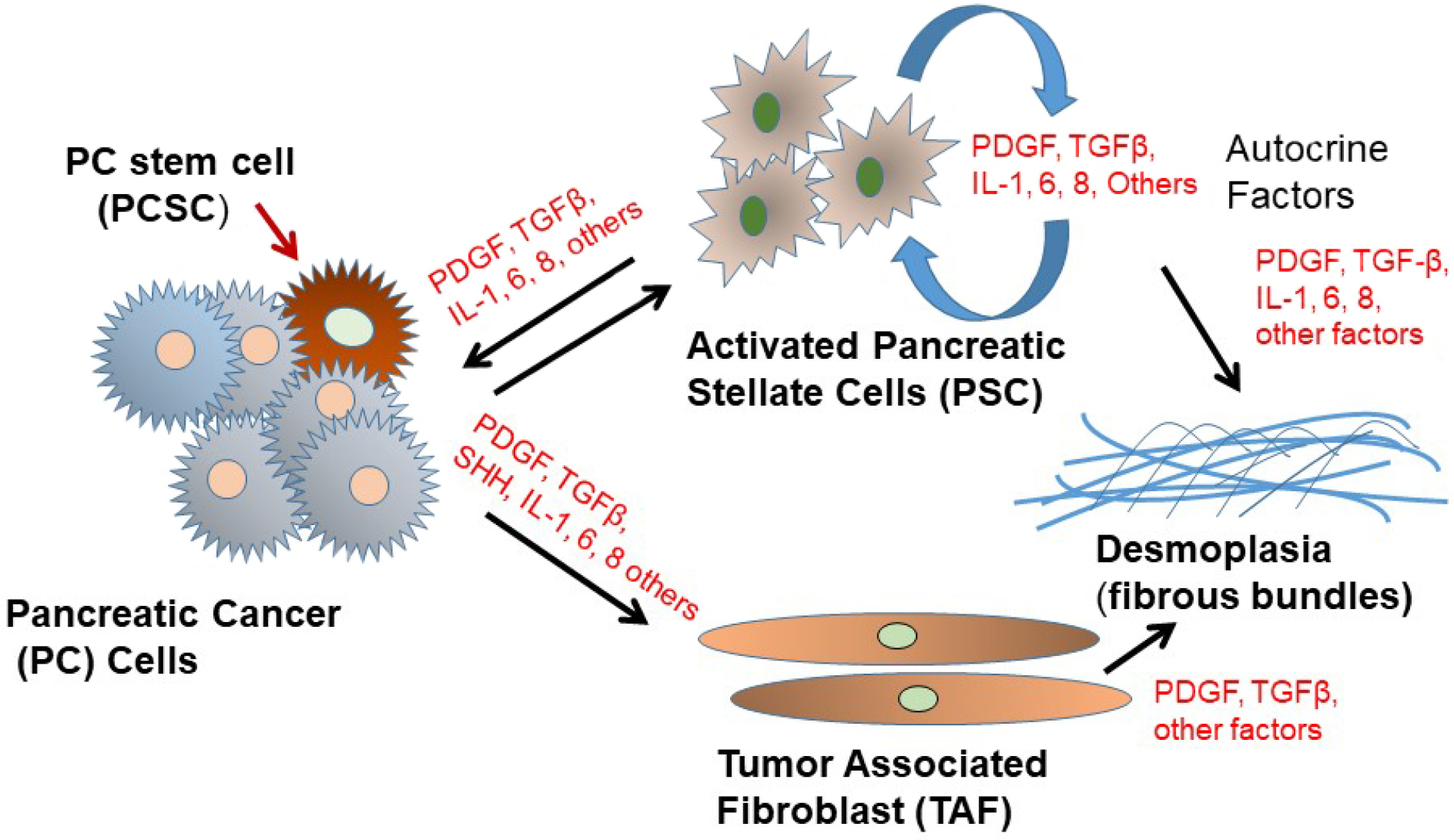
Schematic depiction of pancreatic cancer cell interactions with tumor-associated fibroblasts (TAF) and activated pancreatic stellate cells (PSC) in the tumor microenvironment. PDGF: platelet-derived growth factor; TGFβ: transforming growth factor β

A distinct characteristic of PDAC is its desmoplasia, consisting of a significant amount of cancer-associated fibroblasts (CAFs) and a very dense fibrotic stroma^[51]^ [Figure 2]. The CAFs are pro-inflammatory due to activation of several signaling factors including nuclear factor kappa B (NF-κB), signal transducer and activator of transcription (STAT)-1 and STAT-3, and transforming growth factor (TGF)-β/SMAD^[51–54]^. These signaling factors cooperate in active cross-talk with cancer cells through paracrine signaling factors including chemokines, insulin-like growth factor, and proteases^[52–56]^. Furthermore, several pro-stemness paracrine factors are secreted by distinct CAFs^[56–62]^ and support the self-renewal and the stemness properties of initial PCSCs in tumors or promote the conversion of cancer cells into PCSCs^[63]^. Additionally, chemotherapy (e.g., GEM), can affect CAFs in PDAC, which then acquire a senescence-like secretory phenotype and increase the production of pro-stemness chemokines which enhance tumorstemness and aggressiveness of PDAC after therapy^[13]^. It is known that CAFs consist of a heterogeneous population, with specific functions within tumors and in the process of metastasis^[64]^.

Interestingly, besides CAFs, the PDAC stroma also contains bone marrow-derived mesenchymal stem cells (MSCs)^[65]^. The MSCs significantly contribute to tumor progression and promote cancer stemness by secreting pro-stemness cytokines, chemokines, and growth factors or by differentiating into pro-stemness CAFs^[62,63,66]^. MSCs also produce pro-stemness niches in the stroma of PDAC, and infiltrating immune cells produce pro-stemness factors that form pro-PCSC niches^[67–70]^. Waghray *et al.*^[71]^ identified and characterized mesenchymal stem cells (MSC) within the human PDAC tumor microenvironment (TME). These cancer-associated MSCs (CA-MSCs) increase the growth, invasion, and metastatic potential of PDAC cancer cells^[71]^, and CA-MSCs secrete the cytokine granulocyte-macrophage colony-stimulating factor (GM-CSF) that is required for tumor cell proliferation, invasion, and trans-endothelial migration. The depletion of GM-CSF in CA-MSCs inhibited the ability of these cells to promote tumor cell growth and metastasis^[71]^. Therefore, CA-MSCs may provide a potential strategy for a PDAC therapeutic approach. Since the desmoplastic stroma in PDAC is believed to be a major barrier for the efficient penetration of anti-cancer agents into the tumor, efficient anti-PCSC therapeutics plus anti-stromal or stromal remodeling therapeutics may be able to penetrate through the thick layer of stroma to reach PCSCs and the bulk of tumor cells, to trigger their cell death or growth inhibitory effects and effectively inhibit growth and metastasis of PDAC.

In addition to PCSCs and the previously discussed distinct stromal cells, the PDAC immune system also plays a critical and complex role in the development and progression of PDAC. It is well-established that myeloid-derived suppressor cells (MDSCs) and tumor-associated macrophages (TAMs) alter the immune environment of the TME and help tumor proliferation as well as metastatic and immunotherapy resistance^[72]^. Inhibitors of the CSF-1R were shown to reprogram the TME and TAMs and lead to enhanced T-cell-mediated tumor elimination^[72]^. Furthermore, FAK inhibitors reduced the infiltration of MDSCs, TAMs, and regulatory T-cells^[72]^. Moreover, C-C motif chemokine receptor (CCR)-2 has been shown to mediate the recruitment of TAMs to the tumor^[73]^. Bone marrow mesenchymal stem cells (BM‐MPCs) display self‐renewal, differentiation, dormancy, and hematopoiesis properties; BM‐HPCs also secrete cytokines and extracellular matrix for the growth of metastases^[74–77]^. BM‐MSCs are major players in the tumor microenvironment^[78]^ and affect inflammation, the tumor environment, immunity, and cancer metastasis^[79]^.

### Signaling pathways in PCSCs

**Figure 3.**
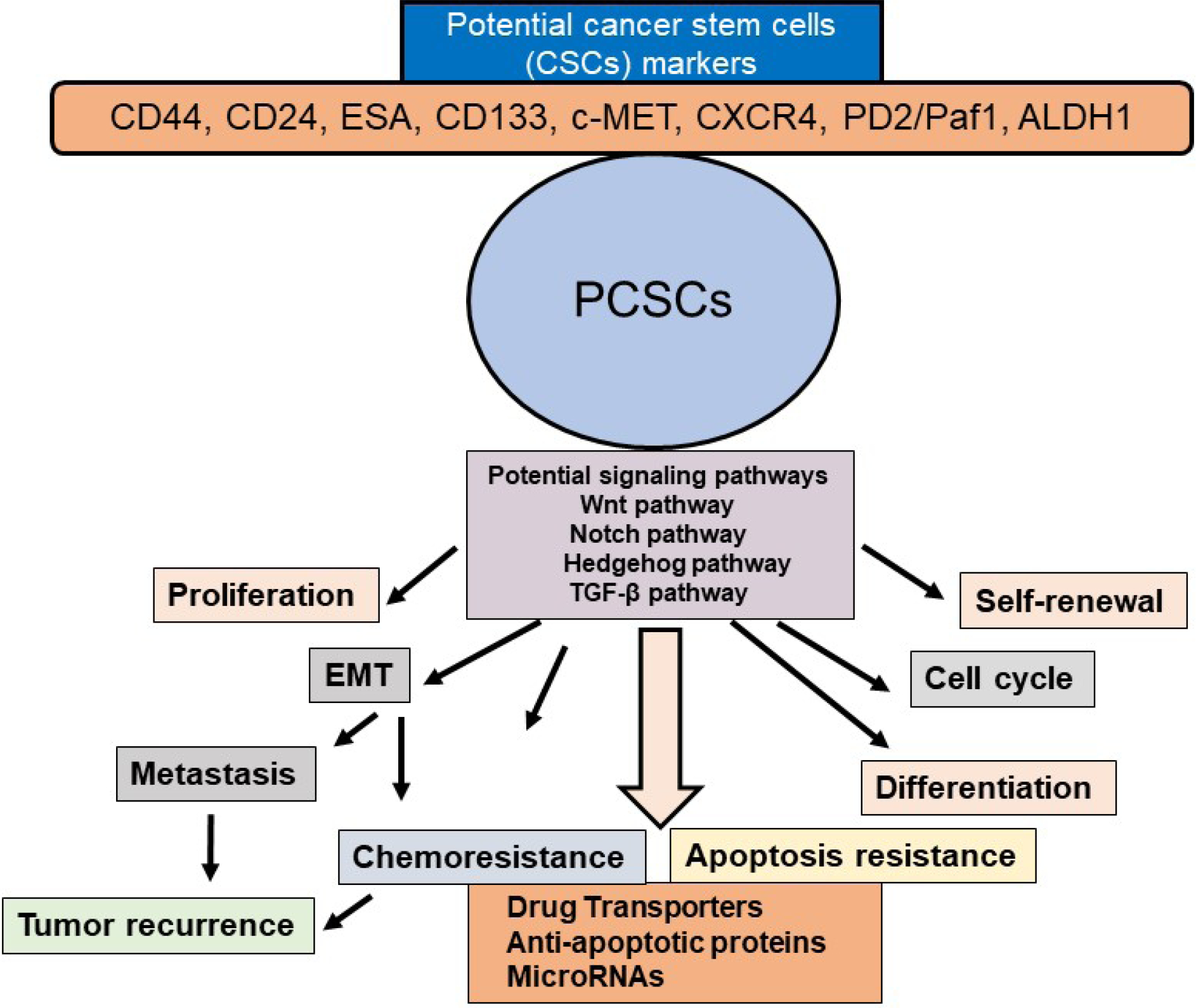
Schematic presentation of PCSC properties and their contribution to EMT, tumor metastasis and recurrence, chemoresistance, and apoptosis resistance. Potential CSC markers and signaling pathways in PCSCs and activation of several signaling pathways in the self-renewal, maintenance, and tumor recurrence in PDAC are also shown. PCSC: pancreatic cancer stem cell; EMT: epithelial-mesenchymal transition

Various signaling pathways are altered in PCSCs and EMT cells including Hedgehog, Notch, Wnt, NF-κB, and AKT [Figure 3]. Among these, Hedgehog, Notch, and Wnt play particularly important roles in PCSCs^[51]^. These signaling pathways are critical regulators of PCSC self-renewal, tumor growth, invasion, metastasis, and therapy resistance^[16,40,80,81]^. Furthermore, miRNAs play a significant role in the regulation of PCSCs^[82,83]^.

Notch signaling regulates cell proliferation, survival, apoptosis, and the differentiation of various cancers including pancreatic cancer cells and PCSCs as well as promoting EMT by controlling some transcription factors and growth factors including Snail, Slug, and TGF-β. Notch targets many genes which play critical roles in the development and progression of human malignancies^[84–86]^. Several studies have demonstrated that resistance to chemotherapy in PCSCs is linked to the active Notch signaling pathway^[63,87,88]^.

Another self-renewal pathway in PCSCs is Hedgehog signaling, which is involved in tumor initiation, progression, and metastasis^[63,89,90]^. The three hedgehog genes include Sonic hedgehog (Shh), Indian hedgehog, and the Desert hedgehog homolog^[90,91]^. It has been shown that one of these three ligands binds to the receptor Patched1 and releases the protein smoothened (Smo)^[63,89,90]^. Smo triggers the activation of downstream target genes such as the GLI family of transcription factors and PTCH. Yamasaki *et al.*^[92]^ have reported that a nine-fold increase in Shh mRNA levels has been found in CD44+CD24+ESA+ PCSCs when compared to the bulk of unsorted pancreatic cancer cells. Inhibition of Hedgehog signaling by Smo suppression has been shown to reverse EMT and suppress the invasion of pancreatic cancer cells^[93–95]^.

Substantial evidence has shown that Wnt/β-catenin signaling is involved in cell proliferation, migration, apoptosis, differentiation, and self-renewal of CSCs in several types of cancers^[96–98]^. Dysregulation of the Wnt/β-catenin signaling pathway is associated with chemotherapy resistance in PDAC, and significant evidence suggests that nuclear β-catenin plays an essential role in EMT^[97,99]^. The Wnt signaling pathway also plays a significant role in regulating PCSCs^[99,100]^. Additionally, Wnt signals significantly regulate CSCs in solid tumors including PDAC in the niche environments^[98,101,102]^. Because of dysregulation in the Wnt signaling pathway, PCSCs are significantly susceptible to Wnt signal inhibitors^[98,99,103,104]^. Hence, Wnt in the PDAC niche of PCSCs offers a critical therapeutic target in PDAC.

A significantly activated signaling pathway in CSCs is the NF-κB pathway; its inhibition triggers the loss of CSC properties^[105,106]^. Interestingly, the CCL21/CCR7 axis known to facilitate metastasis to distant organs promoted the metastasis and survival of CD133+ PCSCs and regulated their metastasis by modulating EMT and the Erk/NF-κB pathway^[107]^. Moreover, NF-κB-mediated invasiveness in CD133+ PCSCs is regulated by autocrine and paracrine activation of IL1 signaling^[108]^. Furthermore, the crucial role of PCSCs in developing resistance to gemcitabine treatment through the Nox/ROS/NF-κB/STAT3 signaling pathway was demonstrated by Zhang *et al.*^[109]^. Significantly, therapeutic targeting of the FGFR1/Src/NF-κB signaling axis has been shown to inhibit PCSCs and oncogenicity^[110]^. These findings will provide new directions for identifying potential targets that regulate NF-κB-mediated invasiveness of PCSCs and can be used to sensitize pancreatic cells to chemotherapy. In addition to the above major signaling pathways, the mTOR pathway has been shown to be essential for the self-renewal of PCSCs^[111,112]^.

Like other cancers, miRNA expression is dysregulated in PDAC^[113,114]^. Two classes of miRNAs play crucial roles in cancer cells, oncogenic miRNAs and tumor suppressor miRNAs^[23,115,116]^. Jung *et al.*^[117]^ demonstrated that PCSCs exhibit differential expression of miR-99a, miR-100, miR-125b, miR-192, and miR-429 compared with controls. Another study reported the loss of miR-34 in CD44+CD133+ PCSCs, while miR-34 restoration led to the inhibition of spheroidal growth of CSCs and tumor formation^[118]^. Wellner *et al.*^[119]^ showed that miR-200c, miR-203, and miR-183 down regulate stem cell factors and described a regulatory feedback loop between miRNAs and CSC in pancreatic cancer. Moreover, they demonstrated that ZEB1 represses expression of stemness-inhibiting miR-203 and that ZEB1 links EMT activation and stemness maintenance by suppressing stemness-inhibiting miRNA expression, and therefore promotes mobile migrating CSCs. These authors concluded that targeting the ZEB1-miR-200 feedback loop may potentially be a valid and promising therapeutic approach for PDAC.

While this review discusses abnormalities in the intracellular signaling pathways known to be involved in carcinogenesis, growth, metastasis, EMT, and chemoresistance in PDAC, the significance of these regulatory pathways to the major risk factors for pancreatic cancer including diabetes, smoking, alcoholism, and psychological stress remains to be discovered.

## EPITHELIAL-MESENCHYMAL (EMT) TRANSITION

A small number of cancer cells in the primary tumor are capable of undergoing EMT, which critically promotes tumor invasion and metastatic dissemination^[120,121]^ in human cancer patients including PDAC^[40,41,122]^, as well as in PCSCs and circulating tumor cells^[122–124]^. Metastatic seeding is frequently initiated before diagnosis of the primary tumor in cancer patients, and disseminated tumor cells can remain dormant in secondary sites before forming metastatic tumors^[125–128]^. The majority of pancreatic cancer-related mortality is due to metastatic disease.

EMT is critical for this rapid metastatic tumor progression, and it is a multi-stage trans-differentiation cellular process that allows epithelial cells to undergo multiple biochemical changes to gain a mesenchymal phenotype^[128,129]^. During this process, epithelial cells lose their epithelial markers (such as E-cadherin, occludin, claudin, and laminin 1) and gain mesenchymal markers such as N-cadherin, vimentin, and fibronectin^[130]^. These changes are caused by the activation of specific EMT transcriptional programs. Some transcriptional regulators such as TWIST, SNAI1, SNAI2, ZEB1, and ZEB2 repress E-cadherin expression, while others play roles in promoting the expression markers of mesenchymal differentiation markers including N- and/or R-cadherin and vimentin, as well as cellular matrix and focal adhesion proteins involved in promoting motility^[124,131,132]^. Furthermore, EMT is associated with enhanced activity of matrix metalloproteinases^[124,131,132]^.

After the invading cancer cells reach metastatic sites, they undergo the reverse EMT process, MET, and adapt to proliferating in the invaded tissue microenvironment^[133–136]^. Since metastasis is the major cause of cancer mortality, a detailed understanding of EMT could potentially lead to more effective therapeutic strategies for PDAC.

## METASTASIS AND CSC POPULATION

Emerging evidence indicates that as the tumor microenvironment opts for a CSC population, these cells can also metastasize and initiate cells that participate in metastasis^[137]^. Several cellular signaling pathways regulate the self-renewal capacity of CSCs and control their inter-conversion between the dormantstate and subsequent re-activation upon metastasis^[137–140]^. These re-activated cells are nurtured by extracellular niches, which crosstalk and support positive cytoprotective signals such as Wnt and Notch^[96,141–145]^. As Mu *et al.*^[15]^ described, tumor progression is driven by the cross-interaction between tumor cells, primarily CSCs and surrounding stromal cells as well as distant organs, in which tumor-derived extracellular vesicles (TEX) play a major and important role. Mu *et al.*^[15]^ report that the PCSC markers Tspan8, alpha6beta4, CD44v6, CXCR4, LRP5/6, LRG5, claudin, EpCAM, and CD133 participate in the metastatic cascade at different stages, often via PDAC TEX. In PDAC tumors, the PCSC population shows over-expression of genes involved in EMT and the distinct ability to metastasize and colonize distant tissues.

CD44 is overexpressed in CSCs, frequently shows alternative spliced variants, and plays a role in cancer development and progression. The CD44 major ligand, hyaluronan, binds to and activates CD44 resulting in stimulation of several cell signaling pathways that trigger cell proliferation, survival, and increased cellular motility^[146]^. Cancer cells that undergo an EMT acquire properties of CSC and show enhanced CD44 expression^[43]^. CD44 consists of two isoforms, standard (CD44s) and CD44 variants(CD44v), but the different functional roles of these variants are not well known. CD44v may play a role in regulating EMT and the plasticity of cancer cells^[146–148]^. Moreover, evidence shows that CD44 activates the MT1-MMP-SNAI1 axis to promote metastasis in PDAC, and CD133 activates the IL1β-NF-κB pathway leading to invasiveness and metastasis^[108,149]^. Interestingly, Zhang *et al.*^[107]^ demonstrated that the CCR7–CCL21 axis in PCSCs mediates EMT phenotype via activation of ERK/NF-κB signaling pathways. Additionally, it has been shown that the Hh pathway is implicated in playing a role in EMT in PCSCs. Inhibition of Hh signaling in CSCs reduced self-renewal, EMT tumorgenesis, invasiveness, drug resistance, and metastasis^[150]^. Activation of the PI3K/AKT/mTOR pathway^[151]^ alone or in combination with the Hh pathway^[152]^ in PCSCs also increased metastasis in these cells.

## RELEVANCE OF PCSCS IN PDAC DEVELOPMENT

The foregoing discussion provided ample evidence related to the role of PCSCs in carcinogenesis, growth, metastasis, EMT, and chemoresistance in PDAC. The above aberrant signaling pathways govern cancer cell plasticity, which give rise to tumor cellular heterogeneity, EMT, therapeutic resistance, and recurrence through clonal replacement and activation of dormant CSCs in PDAC as well as other cancers^[80,88,153,154]^. PDAC is characterized by molecular alterations regulating PCSCs, including mutations of K-RAS, TP53, transforming growth factor-β, Hedgehog, WNT and NOTCH signaling pathways. Many genetic alterations were defined in PDAC such as earlier events including K-ras point mutation^[155]^, INK4a/Arf deficiency^[156]^, the epidermal growth factor receptor (EGFR) over-expression, gene amplification and HER2/neu over-expression and TP53, transforming growth factor-β, Hedgehog, WNT and NOTCH signaling pathways maintain PCSCs. Furthermore, the roles of EGFR, Ras/Raf/MEK/ERK and PI3K/PTEN/Akt/mTORC1/GSK-3 pathways in PCSCs and their relationship to PDAC tumor initiation, EMT, malignant development has been investigated^[157]^. As discussed above, recent advances in the role of EMT and PCSCs in tumor progression, metastasis, chemo-resistance, and the mechanisms integrated with aberrant biochemical signals and the underlying pathways have been surveyed in this review. Furthermore, WNT signaling cascades cross-interaction with the FGF, Notch, Hedgehog and TGFβ/BMP signaling cascades and regulate expression of functional CSC markers, such as CD44, CD133 (PROM1), EPCAM and LGR5 (GPR49)^[40,153–158]^. Aberrant canonical and non-canonical WNT signaling in human malignancies, including PDAC are involved in CSC survival, bulk-tumor expansion and invasion/metastasis^[158]^. Despite these advances, the significance of these regulatory pathways to the major risk factors for pancreatic cancer including diabetes, smoking, alcoholism, and psychological stress remain to be studied in detail in the future.

## CONCLUSION

Substantial evidence has demonstrated that CSCs including PCSCs trigger the characteristic hallmarks of various tumors including self-renewal, invasiveness, tumor recurrence, resistance, metastasis, and resistance to chemotherapeutic agents and radiotherapy. Moreover, the bulk of cancer cell population displays plasticity in most tumors including PDAC, which enables them to dynamically inter-convert between non-CSC and CSC states. Another critically important and intriguing characteristic of CSCs including PCSCs is their capacity to disseminate, migrate, and form metastatic lesions expressing resistance to therapies. In some tumors including PDAC, this plasticity has been associated with the EMT process. Furthermore, cytokines and growth factors, provided by the CSC niche containing CAFs, MSCs, endothelial cells and specific immune cells, and hypoxia, trigger transcriptional and epigenetic regulations leading to the induction of plasticity, stemness, EMT, and metastasis. Taken together, the foregoing discussion in this review provides a better understanding of the molecular mechanisms underlying these behaviours in CSCs, including PCSCs, and may lead to the identification of specific therapeutics and novel strategies to prevent EMT and metastasis, trigger CSC growth inhibition and cell death, and increase the sensitivity of tumors including PDAC to cancer therapeutics.
